# Burden of obesity in the Eastern Mediterranean Region: findings from the Global Burden of Disease 2015 study

**DOI:** 10.1007/s00038-017-1002-5

**Published:** 2017-08-03

**Authors:** GBD 2015 Eastern Mediterranean Region Obesity Collaborators, Charbel El Bcheraoui, Ashkan Afshin, Raghid Charara, Ibrahim Khalil, Maziar Moradi-Lakeh, Nicholas J. Kassebaum, Michael Collison, Farah Daoud, Kristopher J. Krohn, Adrienne Chew, Stan H. Biryukov, Leslie Cornaby, Kyle J. Foreman, Michael Kutz, Patrick Liu, Marissa Reitsma, Patrick Sur, Haidong Wang, Ben Zipkin, Johan Ärnlöv, Cristiana Abbafati, Abdishakur M. Abdulle, Niveen M. E. Abu-Rmeileh, Muktar Beshir Ahmed, Ziyad Al-Aly, Khurshid Alam, Reza Alizadeh-Navaei, Ala’a Alkerwi, Khalid A. Altirkawi, Nelson Alvis-Guzman, Bernhard T. Baune, Neeraj Bedi, Derrick A. Bennett, Zulfiqar A. Bhutta, Mulugeta M. Birhanu, Hadi Danawi, Seyed-Mohammad Fereshtehnejad, Tsegaye Tewelde Gebrehiwot, Paramjit Singh Gill, Philimon N. Gona, Vipin Gupta, Tesfa Dejenie Habtewold, Randah Ribhi Hamadeh, Samer Hamidi, Habtamu Abera Hareri, Masako Horino, Mohamed Hsairi, Mehdi Javanbakht, Denny John, Jost B. Jonas, Vasna Joshua, Yousef Saleh Khader, Ejaz Ahmad Khan, Jagdish Khubchandani, Yun Jin Kim, Yohannes Kinfu, Yoshihiro Kokubo, Heidi J. Larson, Paul H. Lee, Raimundas Lunevicius, Hassan Magdy Abd El Razek, Mohammed Magdy Abd El Razek, Reza Malekzadeh, Kedar K. Mate, Ziad A. Memish, Mubarek Abera Mengistie, George A. Mensah, Felix Akpojene Ogbo, Farshad Pourmalek, Anwar Rafay, Mostafa Qorbani, Vafa Rahimi-Movaghar, Saleem M. Rana, Salman Rawaf, Andre M. N. N. Renzaho, Satar Rezaei, Mohammad Sadegh Rezai, Mahdi Safdarian, Mohammad Ali Sahraian, Payman Salamati, Juan Ramon Sanabria, Milena M. Santric Milicevic, Benn Sartorius, Sadaf G. Sepanlou, Masood Ali Shaikh, Rahman Shiri, Diego Augusto Santos Silva, Jasvinder A. Singh, Abdullah Sulieman Terkawi, Tenaw Yimer Tiruye, Kingsley Nnanna Ukwaja, Vasiliy Victorovich Vlassov, Tolassa Wakayo, Andrea Werdecker, Naohiro Yonemoto, Mustafa Z. Younis, Bassel Zein, Aisha O. Jumaan, Theo Vos, Simon I. Hay, Mohsen Naghavi, Christopher J. L. Murray, Ali H. Mokdad

**Affiliations:** Institute for Health Metrics and Evaluation, University of Washington, Seattle, WA USA

**Keywords:** Obesity, Burden of disease, Eastern Mediterranean Region

## Abstract

**Objectives:**

We used the Global Burden of Disease (GBD) 2015 study results to explore the burden of high body mass index (BMI) in the Eastern Mediterranean Region (EMR).

**Methods:**

We estimated the prevalence of overweight and obesity among children (2–19 years) and adults (≥20 years) in 1980 and 2015. The burden of disease related to high BMI was calculated using the GBD comparative risk assessment approach.

**Results:**

The prevalence of obesity increased for adults from 15.1% (95% UI 13.4–16.9) in 1980 to 20.7% (95% UI 18.8–22.8) in 2015. It increased from 4.1% (95% UI 2.9–5.5) to 4.9% (95% UI 3.6–6.4) for the same period among children. In 2015, there were 417,115 deaths and 14,448,548 disability-adjusted life years (DALYs) attributable to high BMI in EMR, which constitute about 10 and 6.3% of total deaths and DALYs, respectively, for all ages.

**Conclusions:**

This is the first study to estimate trends in obesity burden for the EMR from 1980 to 2015. We call for EMR countries to invest more resources in prevention and health promotion efforts to reduce this burden.

**Electronic supplementary material:**

The online version of this article (doi:10.1007/s00038-017-1002-5) contains supplementary material, which is available to authorized users.

## Introduction

High body mass index (BMI), or overweight, is associated with increased morbidity and mortality, and is a major risk factor for diabetes, cancer, cardiovascular diseases, sleep apnea, and poor physical health (Kim et al. 2016; Yao et al. 2017; Kelly et al. 2017; Mehta et al. 2017). It is also associated with an increased risk for psychiatric disorders, including depression (Pratt and Brody 2014; Abou Abbas et al. 2015). A continuous rise in obesity is threatening health improvements in many countries (Sidney et al. 2016; GBD 2015 Obesity Collaborators 2017), while controlling its spread could drastically improve population health (Maciosek et al. 2017).

The increase in overweight and obesity prevalence is a direct result of lifestyle changes due to the social and demographic transition that started several decades ago (Broyles et al. 2015; Mokdad et al. 2016). The Eastern Mediterranean Region (EMR) is facing the same challenges due to rapid economic, demographic, and lifestyle changes, including changes in food consumption, reduced physical activity, and increased sedentary lifestyle (Musaiger et al. 2012; Mokdad et al. 2016). The contribution of high BMI to total DALYs in the EMR had increased in 2013 (7.5% of DALYs) in comparison to 1990 (3.7% of DALYs) (Mokdad et al. 2016). Kuwait, Qatar, and Libya, three EMR countries, were among the top ten countries with highest prevalence of obesity worldwide in 2013, and the man believed to be the heaviest living person was diagnosed in Saudi Arabia (Ng et al. 2014; Terkawi et al. 2014).

The EMR has a population of about 583 million people (World Health Organization 2016). Countries in the EMR vary significantly in terms of their gross domestic product, socio-demographic profiles, health indicators, and health system capacities and coverage. Despite the heavy burden of high BMI in the region, no comprehensive and current estimates of the epidemic exist for the EMR.

To quantify the burden of high BMI in the EMR and its impact on health, we systematically evaluated the trends in prevalence of overweight and obesity as well as the patterns of deaths and DALYs related to high BMI by age and sex, using the results of the Global Burden of Disease (GBD) 2015 study. We also estimated the attributable fraction of high BMI to ischemic heart disease, stroke, and diabetes mellitus, the three leading non-communicable causes of death in EMR, for which high BMI is a risk factor.

## Methods

The prevalence of overweight and obesity among children (2–19 years) and adults (≥20 years) in 1980 and 2015 was estimated for EMR countries. The EMR countries, based on the World Health Organization classification, are Afghanistan, the Kingdom of Bahrain, Djibouti, the Arab Republic of Egypt, the Islamic Republic of Iran, the Republic of Iraq, the Hashemite Kingdom of Jordan, the State of Kuwait, the State of Lebanon, the State of Libya, the Kingdom of Morocco, the Sultanate of Oman, the Islamic Republic of Pakistan, Palestine, the State of Qatar, the Kingdom of Saudi Arabia, the Federal Republic of Somalia, the Republic of Sudan, the Syrian Arab Republic, the Republic of Tunisia, the United Arab Emirates, and the Republic of Yemen.

The burden of disease related to high BMI was calculated using the GBD comparative risk assessment approach between 1990 and 2015 (Forouzanfar et al. 2015, 2016). A detailed methodology of BMI estimation for GBD 2015 has been published elsewhere (GBD 2015 Obesity Collaborators 2017). Since burden estimations depend on GBD all-cause mortality, burden of high BMI is only available for the period 1990–2015. We used all available data surveys following a systematic search. The search strategy as well as data sources used per country have been published as an appendix elsewhere and are available from the Global Health Data Exchange (Institute for Health Metrics and Evaluation 2016; GBD 2015 Obesity Collaborators 2017). Briefly, Medline was systematically searched for studies providing nationally or subnationally representative estimates of overweight prevalence, obesity prevalence, or mean body mass index (BMI) published between 1 January 2014 and 31 December 2015 to update the GBD 2013 systematic literature search (Ng et al. 2016).

For adults, 127 out of 2036 abstracts identified met inclusion criteria and were extracted. For children, 146 out of 971 articles identified were extracted. In total, 816 articles were included in the analysis. Additionally, the Global Health Data Exchange (GHDx) database was searched for individual-level data from major multinational survey series or country-specific surveys and identified 1026 unique sources meeting the inclusion criteria. Of the 816 articles and 1026 unique sources, all those pertaining to EMR countries were included. The GBD 2015 results tool from the GHDx allows readers to view, country by country, what data sources have been used to produce these estimates (Institute for Health Metrics and Evaluation 2016).

For adults, overweight was defined as 25.0 ≤ BMI ≤ 30 kg/m^2^, and obesity was defined as BMI ≥30 kg/m^2^. The International Obesity Task Force definition was used for childhood overweight and obesity (Cole et al. 2000).

Children were defined as individuals 2–19 years of age based on the lowest age for which the International Obesity Task Force provides a definition of overweight and obesity, and the age groups used in GBD modeling, which include 19 in the age group 15–19 (Cole et al. 2000; Wang et al. 2016).

Briefly, a spatiotemporal Gaussian process regression (ST-GPR) was used to estimate the mean prevalence of overweight and obesity (Ng et al. 2014). To improve estimates for countries with sparse data, three country-level covariates with best fit and coefficients in the expected direction were selected: 10-year lag-distributed energy intake per capita, the absolute latitude of the country as proxy for income, and the proportion of people living in urban areas. These covariates have been systematically evaluated in a previous study (Ng et al. 2014).

The Bradford Hill criteria for causation and the World Cancer Research Fund evidence grading criteria were used to systematically evaluate epidemiologic evidence supporting the causal relationship between high BMI and various diseases among adults (≥20 years of age) (Hill 1965; World Cancer Research Fund and American Institute for Cancer Research 2007).

The population-attributable fraction by country, age, sex, and year was calculated to quantify the burden of disease related to high BMI, defined as BMI ≥25 kg/m^2^, for each disease. Deaths and DALYs related to high BMI for each country, age, sex, year, and cause were computed by multiplying the population-attributable fraction by the total deaths or DALYs estimated in GBD 2015 for that country, age, sex, year, and cause. The total disease burden of high BMI was calculated as the sum of disease-specific burden.

95% uncertainty intervals (UI) for all results were computed using Monte Carlo simulations, keeping 1000 draws of each quantity of interest to propagate uncertainty into final estimates.

Expected estimates were also produced for each country based on its Socio-demographic Index (SDI)—a summary measure of lag-distributed income per capita, average educational attainment over the age of 15 years, and total fertility rate (Forouzanfar et al. 2015). In GBD 2015, SDI was computed by rescaling each component to a scale of zero to one, with zero being the lowest observed educational attainment, lowest income per capita, and highest fertility rate from 1980 to 2015, and one being the highest observed educational attainment, highest income per capita, and lowest fertility rate during that time, and then taking the geometric mean of these values for each location-year.

This study followed the Guidelines for Accurate and Transparent Health Estimates Reporting (GATHER) of the World Health Organization (WHO) regarding documentation of data sources, estimation methods, and statistical analysis (Stevens et al. 2016).

### Role of the funding source

The Bill & Melinda Gates Foundation had no role in the development of these methods.

## Results

**Fig. 1:**
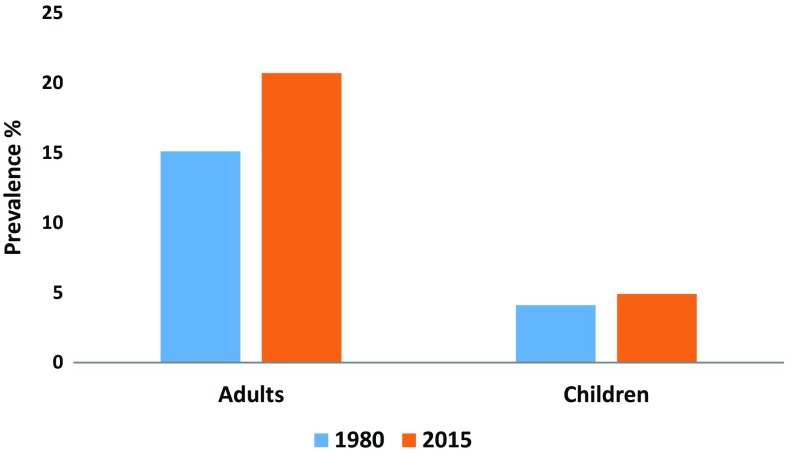
Prevalence of obesity among adults aged 20 years or older and children aged 2–19 years in 1980 and 2015 (Global Burden of Disease 2015 study, Eastern Mediterranean Countries, 1980 and 2015)

The mean BMI increased from 25.2 kg/m^2^ [95% uncertainty interval (UI) 24.9–25.5] in 1980 to 26.0 kg/m^2^ (95% UI 25.8–26.3) in 2015 in the EMR among persons aged 20 years or older. The prevalence of obesity increased from 15.1% (95% UI 13.4–16.9) to 20.7% (95% UI 18.8–22.8) for the same period and for the same age group (Fig. 1). It increased from 4.1% (95% UI 2.9–5.5) to 4.9% (95% UI 3.6–6.4) for the same period among those aged 2–19 years (Fig. 1). The highest prevalence of obesity among adults 20 years or older in 2015 was observed in Qatar: 42.5% (95% UI 40.1–44.8) for males and 52.4% (95% UI 50.3–54.5) for females (e-Table 1); and the highest for children 2–19 years was observed in Kuwait: 22.1% (95% UI 17.8–27.0) for males and 19.2% (95% UI 15.2–23.4) for females (e-Table 2). The lowest prevalence of obesity was observed in Somalia among individuals 20 years or older: 2.5% (95% UI 1.5–4.0) for males and 10.5% (95% UI 8.3–12.8) for females (e-Table 1), and in Yemen and Pakistan for children 2–19 years of age: 1.3% (95% UI 0.9–1.8) for males in Yemen and 2.2% (95% UI 1.4–3.3) for females in Pakistan (e-Table 2). Prevalence of obesity was higher in females than males 20 years or older for all countries, with Sudan having the highest difference between sexes: 11.4% (95% UI 10.0–13.1) for males and 28.3% (95% UI 25.6–31.2) for females (e-Table 1). The highest difference in obesity prevalence between sexes for children was observed in Qatar: 20.8% (95% UI 16.5–25.1) for males and 13.5% (95% UI 10.3–17.1) for females. In children, prevalence of obesity was higher in males for several countries (e-Table 2).

### Deaths

In 2015, there were 417,115 deaths attributable to high BMI in EMR, which constitute about 10% of total deaths in the region for all ages. This is a rate of 120.1 (95% UI 87.5–156.2) deaths per 100,000 population, an 11% increase since 1990. It contributed to 5.0, 0.9, and 1.9% of all deaths cause by ischemic heart disease, ischemic stroke, and diabetes mellitus, respectively. Contribution by specific age groups is detailed in Table 1. In 2015, the rate of deaths attributable to high BMI was highest in Afghanistan, 227.6 (95% UI 146.2–319.5), and lowest in Tunisia, 64.8 (95% UI 42.7–92.5) per 100,000 population (Table 2). Overall, death rates attributable to high BMI have increased in 11 countries and decreased in 11 (Table 2). The largest increase in deaths per 100,000 population attributable to high BMI was observed in Djibouti: from 37.3 (95% UI 15.1–70.6) in 1990 to 101.1 (95% UI 47.5–192.7) in 2015 (Table 2). The largest decrease was observed in Lebanon: from 124.8 (95% UI 85.2–169.7) in 1990 to 72.5 (95% UI 47.0–101.0) in 2015 (Table 2).

**Table 1:** Deaths, with 95% uncertainty intervals (UI), per 100,000 population attributable to high body mass index among those who died from ischemic heart disease, stroke, and diabetes, by age groups (Global Burden of Disease 2015 study, Eastern Mediterranean countries, 1990 and 2015)

Age	Cause	1990		2015	
		Males	Females	Males	Females
15–49	Ischemic heart disease	10.1 (6.0–14.7)	7.0 (4.5–9.5)	12.1 (7.6–17.2)	6.9 (4.7–9.3)
	Stroke	7.1 (4.6–9.8)	8.1 (5.8–10.6)	7.2 (4.7–9.9)	7.3 (5.3–9.6)
	Diabetes	1.7 (1.2–2.3)	2.0 (1.5–2.6)	2.6 (1.8–3.3)	2.9 (2.2–3.6)
50–69	Ischemic heart disease	118.4 (67.9–177.4)	118.1 (80.0–161.8)	134.4 (82.3–191.6)	107.1 (74.8–142.7)
	Stroke	58.1 (33.6–85.1)	77.8 (53.9–107.7)	60.6 (38.4–85.5)	68.7 (49.1–91.2)
	Diabetes	24.8 (15.7–34.4)	36.4 (25.1–50.1)	39.2 (27.2–52.3)	53.4 (40.6–67.8)
70+	Ischemic heart disease	283.8 (141.5–460.9)	366.1 (225.5–539.6)	336.9 (183.3–532.9)	361.0 (222.4–520.7)
	Stroke	117.4 (58.4–198.2)	163.6 (96.9–248.3)	125.8 (65.8–203.8)	150.7 (89.1–223.9)
	Diabetes	57.7 (30.8–89.2)	81.9 (47.6–130.2)	106.5 (59.1–163.1)	160.3 (103.1–224.8)

**Table 2:** Deaths, with 95% uncertainty intervals (UI), per 100,000 population due to high body mass index observed in 1990 and 2015, and expected based on Socio-demographic Index in 2015 (Global Burden of Disease 2015 study, Eastern Mediterranean countries, 1990 and 2015)

Country	1990 deaths		2015 deaths		Total observed	Total expected
	Males	Females	Males	Females		
Afghanistan	166.9 (80.9–283.8)	296.3 (170.9–460.1)	160.5 (78.6–268.2)	292.1 (166.4–450.7)	27,827.4 (17,910.5–39,914.8)	5797.5
Bahrain	133.2 (84.8–191.7)	175.2 (129.1–222.4)	97.7 (62.1–141.3)	99.2 (68.9–130.4)	557.5 (421.6–709.1)	443.3
Djibouti	37.1 (9.8–94.0)	36.7 (11.8–82.8)	102.0 (35.3–229.3)	99.1 (37.7–231.1)	467.9 (211.4–929.9)	312.5
Egypt	153.2 (98.2–207.3)	154.4 (116.9–192.2)	176.2 (123.4–228.0)	154.6 (121.5–188.6)	90,774.1 (73,283.6–108,972.5)	48,851.2
Iran	90.7 (44.8–146.5)	98.9 (63.3–138.2)	102.3 (56.9–162.0)	94.4 (59.8–137.7)	49,386.0 (33,333.8–68,369.9)	44,524.9
Iraq	234.4 (158.3–335.3)	243.7 (171.0–324.3)	217.4 (129.1–321.1)	206.3 (136.0–295.7)	30,963.7 (22,483.9–41,095.5)	12,627.7
Jordan	144.8 (93.7–209.6)	198.5 (146.6–258.2)	118.8 (80.1–160.0)	107.3 (80.4–136.1)	3687.0 (2946.9–4478.1)	3082.9
Kuwait	92.2 (64.5–120.0)	115.1 (89.1–142.1)	93.9 (65.2–126.1)	93.6 (70.6–120.7)	1335.8 (1071.0–1657.8)	670.7
Lebanon	127.0 (70.4–198.5)	122.6 (76.9–179.0)	69.4 (35.3–113.1)	75.2 (44.8–109.8)	3565.9 (2320.6–4940.1)	3803.3
Libya	81.8 (49.9–120.0)	103.5 (72.0–137.6)	96.8 (59.6–141.4)	109.7 (78.2–148.0)	3727.3 (2795.3–4725.7)	3261.4
Morocco	85.0 (45.4–132.0)	103.3 (65.8–148.5)	77.4 (40.8–129.9)	102.6 (57.3–158.6)	22,383.7 (14,985.5–31,865.0)	18,621.5
Oman	82.0 (46.3–131.0)	89.4 (51.9–140.9)	92.3 (56.9–131.3)	93.4 (63.0–123.8)	1575.4 (1161.4–2011.7)	1619.5
Pakistan	54.3 (22.0–98.4)	67.1 (33.2–113.4)	105.9 (169.5–54.7)	110.6 (64.32–166.3)	110,546.2 (72,034.6–153,911.6)	67,064.8
Palestine	114.4 (63.3–182.1)	128.0 (77.6–197.6)	149.7 (86.8–225.0)	118.2 (77.7–170.3)	2347.3 (1655.2–3168.4)	1502.5
Qatar	142.7 (101.7–187.8)	209.5 (161.3–263.6)	106.6 (69.3–154.4)	126.1 (88.2–169.1)	567.3 (424.8–747.7)	479.1
Saudi Arabia	75.2 (46.7–107.1)	81.9 (59.0–108.4)	84.5 (57.9–114.5)	68.3 (51.6–87.4)	10,888.8 (8682.8–13,471.6)	11,340.5
Somalia	45.3 (8.4–127.5)	107.8 (24.8–251.0)	43.5 (9.5–112.7)	101.0 (24.2–242.5)	3058.6 (722.6–7430.6)	1761.52
Sudan	96.5 (47.9–163.1)	158.6 (95.9–242.4)	101.0 (48.2–171.3)	146.7 (80.2–230.8)	21,814.0 (14,145.4–31,022.9)	10,515.2
Syria	141.1 (88.4–207.5)	145.8 (99.7–205.0)	112.7 (67.3–164.2)	99.3 (67.9–131.0)	9987.0 (7397.8–12,880.3)	8109.9
Tunisia	72.6 (37.9–111.7)	75.4 (49.4–105.8)	71.9 (37.2–118.8)	58.4 (34.7–87.3)	6256.6 (4163.4–8889.9)	8682.7
United Arab Emirates	128.6 (76.3–188.5)	153.1 (100.1–214.7)	103.9 (60.2–156.2)	103.8 (68.8–148.4)	4460.4 (2941.6–6206.4)	1648.5
Yemen	74.5 (27.2–149.7)	106.8 (43.0–205.8)	78.1 (31.1–158.7)	124.4 (59.2–225.4)	10,936.6 (5668.1–19,184.0)	5848.1

### DALYs

In 2015, there were 14,448,548 DALYs attributable to high BMI in the EMR, which constitutes about 6.3% of total DALYs in the region for all ages. This is a rate of 3452.5 (95% UI 2599.2–4386.5) DALYs per 100,000 population, a 13.9% increase since 1990. It contributed to 3.0, 0.5, and 2.3% of all DALYs caused by ischemic heart disease, stroke, and diabetes mellitus, respectively. Contribution by specific age groups is detailed in Table 3. In 2015, the rate of DALYs attributable to high BMI was highest in Afghanistan, 6576.7 (95% UI 4366.1–9219.6), and lowest in Tunisia, 2022.4 (95% UI 1395.1–2724.3) per 100,000 population. Trends in DALYs followed trends in deaths from high BMI in all countries. The largest increase in DALYs per 100,000 population attributable to high BMI was observed in Djibouti: from 1111.0 (95% UI 492.8–2005.8) in 1990 to 2810.8 (95% UI 1432.5–5212.9) in 2015 (Table 4). The largest decrease was observed in Lebanon: from 3552.4 (95% UI 2508.2–4645.4) in 1990 to 2363.8 (95% UI 1707.9–3077.2) in 2015.

**Table 3:** Disability-adjusted life years (DALYs) and years lived with disability (YLDs), with 95% uncertainty intervals (UI), per 100,000 population attributable to high body mass index among DALYs and YLDs due to ischemic heart disease, stroke, and diabetes, by age groups (Global Burden of Disease 2015 study, Eastern Mediterranean countries, 1990 and 2015)

Age	Cause	DALYs				YLDs			
		1990		2015		1990		2015	
		Males	Females	Males	Females	Males	Females	Males	Females
15–49	Ischemic heart disease	473.0 (278.3–690.8)	328.0 (211.6–453.7)	560.1 (352.0–801.7)	327.5 (222.9–436.6)	5.9 (3.0–9.7)	5.3 (3.0–8.5)	9.9 (5.5–15.9)	8.9 (5.3–13.7)
	Stroke	351.1 (225.4–483.7)	398.8 (286.0–519.7)	353.0 (234.5–484.9)	361.8 (261.7–468.2)	12.7 (7.0–20.1)	16.8 (10.5–24.9)	16.8 (9.9–25.6)	22.2 (14.2–31.4)
	Diabetes	251.7 (163.0–351.2)	299.3 (216.5–399.8)	419.6 (284.2–577.4)	483.5 (351.3–640.4)	173.6 (103.8–262.9)	206.7 (133.2–297.0)	303.4 (187.6–448.4)	351.3 (229.4–491.8)
50–69	Ischemic heart disease	3357.1 (1918.0–5002.9)	3270.6 (2220.9–4466.6)	3898.1 (2392.9–5519.0)	3014.3 (2122.1–3987.9)	74.0 (35.4–126.3)	73.6 (41.8–114.4)	116.6 (60.1–192.0)	105.3 (61.8–157.8)
	Stroke	1686.2 (990.7–2460.1)	2247.9 (1556.8–3081.0)	1792.3 (1132.3–2514.2)	2015.4 (1440.7–2648.3)	64.4 (32.9–106.1)	87.3 (53.5–133.0)	81.1 (45.0–126.4)	99.5 (62.9–146.0)
	Diabetes	1471.0 (909.2–2097.8)	2091.7 (1497.4–2815.7)	2417.9 (1604.3–3312.3)	3190.4 (2388.5–4121.2)	787.0 (431.0–1229.8)	1098.0 (689.5–1605.4)	1330.1 (769.0–1994.0)	1736.2 (1119.0–2482.3)
70+	Ischemic heart disease	4001.4 (2028.0–6420.1)	4951.1 (3092.1–7158.9)	4600.8 (2513.9–7234.6)	4690.5 (2977.1–6737.5)	92.0 (42.0–167.8)	110.2 (61.8–180.6)	138.4 (66.9–240.6)	155.1 (88.7–248.2)
	Stroke	1789.7 (919.0–2943.9)	2449.0 (1480.2–3678.2)	1875.5 (991.2–2972.6)	2200.4 (1362.7–3160.4)	60.1 (27.2–106.5)	78.8 (43.8–128.7)	72.2 (34.3–123.3)	90.8 (53.2–142.7)
	Diabetes	1342.6 (725.2–2113.3)	1933.4 (1197.4–2848.9)	2345.0 (1336.8–3535.0)	3392.0 (2234.9–4736.3)	532.3 (258.2–920.5)	802.8 (468.0–1261.9)	921.4 (471.6–1515.4)	1307.7 (782.1–1998.0)

### Expected versus observed

Overall, and based on an SDI of 0.55, a death rate of 74.0 and a DALYs rate of 2114.3 per 100,000 population were expected for the EMR in 2015 for high BMI. Both are lower than the observed death rate of 120.1 (95% UI 87.5–156.2) and DALYs rate of 3452.5 (95% UI 2599.2–4386.5) for the same year. Expected estimates for each country are detailed in Tables 3 and 4.

**Table 4:** Disability-adjusted life years, with 95% uncertainty intervals (UI) per 100,000 population due to high body mass index observed in 1990 and 2015, and expected based on Socio-demographic Index in 2015 (Global Burden of Disease 2015 study, Eastern Mediterranean countries, 1990 and 2015)

Country	1990		2015		Total observed	Total expected
	Males	Females	Males	Females		
Afghanistan	4767.5 (2400.1–7893.8)	8437.9 (4934.2–12,974.6)	4677.5 (2411.1–7598.2)	8491.9 (5022.7–13,139.8)	965,879.5 (648,967.1–1,371,214.6)	206,389.0
Bahrain	4039.9 (2769.8–5453.6)	4907.8 (3800.7–6091.1)	3109.6 (2127.8–4224.1)	3177.8 (2414.8–4028.7)	29,225.3 (22,628.0–36,438.7)	18,576.5
Djibouti	1189.7 (362.1–2830.1)	1023.3 (378.0–2220.0)	2974.8 (1138.0–6633.1)	2633.4 (1192.2–5883.4)	15,630.0 (7793.1–29,440.4)	10,761.1
Egypt	4317.3 (2880.3–5717.9)	4251.7 (3367.6–5153.0)	5025.6 (3688.3–6374.7)	4435.2 (3623.2–5284.9)	3,037,204.4 (2,478,932.1–3,605,791.4)	1,666,166.5
Iran	2509.4 (1318.4–3910.9)	2733.1 (1877.6–3632.2)	2846.6 (1712.4–4306.3)	2696.7 (1880.0–3776.6)	1,698,192.1 (1,199,771.9–2,259,245.3)	1,493,421.0
Iraq	6502.5 (4537.2–8970.9)	6404.8 (4660.0–8287.8)	6321.0 (4108.7–9096.4)	5726.9 (4046.8–7892.3)	1,090,796.9 (821,609.9–1,421,193.6)	452,426.9
Jordan	4299.2 (2938.7–5943.2)	5362.9 (4166.0–6773.0)	3633.7 (2593.7–4668.3)	3176.4 (2469.7–3918.3)	141,869.1 (112,808.2–172,138.2)	106,862.3
Kuwait	2861.0 (2115.1–3562.8)	3229.1 (2627.2–3868.5)	3026.5 (2239.0–3850.2)	2715.3 (2167.4–3389.3)	69,567.5 (55,117.3–84,812.6)	32,170.1
Lebanon	3664.5 (2191.8–5471.8)	3454.1 (2306.5–4827.3)	2251.5 (1351.6–3307.2)	2480.0 (1658.6–3401.0)	123,560.0 (89,344.0–160,488.7)	110,981.5
Libya	2379.4 (1551.1–3309.5)	3037.5 (2274.4–3882.7)	2904.3 (1926.1–3953.5)	3285.7 (2464.7–4171.7)	139,878.3 (108,615.2–173,319.3)	116,450.8
Morocco	2490.8 (1420.6–3677.6)	2952.1 (1987.8–4068.0)	2336.9 (1332.7–3654.0)	3032.9 (1949.9–4325.84	774,701.5 (555,295.0–1,031,236.8)	600,106.4
Oman	2471.6 (1506.0–3723.1)	2805.3 (1835.8–4077.2)	2927.1 (1965.5–3972.0)	3097.2 (2308.7–3934.5)	75,807.5 (57,042.8–96,463.6)	64,799.1
Pakistan	1543.7 (657.2–2752.2)	1869.4 (995.8–3001.1)	3009.9 (1619.5–4710.8)	3083.7 (1950.6–4398.8)	3,608,879.0 (2,420,204.5–4,972,204.0)	2,240,433.8
Palestine	3235.1 (1896.7–4980.2)	3467.5 (2290.6–5022.7)	4300.8 (2694.9–6204.2)	3206.5 (2240.2–4368.5)	82,929.2 (61,735.5–109,791.4)	54,335.0
Qatar	4074.6 (3099.9–5097.3)	5538.0 (4495.6–6656.9)	3236.7 (2297.7–4272.4)	3669.8 (2825.1–4589.9)	36,660.9 (27,986.6–46,001.8)	23,177.6
Saudi Arabia	2296.3 (1512.6–3138.8)	2558.8 (1946.2–3250.2)	2507.0 (1808.4–3262.0)	2165.4 (1693.7–2699.8)	490,128.6 (383,013.6–601,300.7)	440,497.5
Somalia	1463.9 (330.5–3897.1)	2980.2 (780.2–6721.4)	1325.4 (330.3–3416.1)	2828.7 (834.7–6943.8)	102,092.6 (31,197.6–246,633.4)	59,469.2
Sudan	2883.9 (1513.0–4693.9)	4378.6 (2790.2–6417.8)	3057.9 (1567.6–4893.5)	4128.4 (2480.3–6203.9)	764,380.1 (523,579.9–1,052,574.8)	364,535.7
Syria	4198.1 (2692.6–5980.2)	4090.6 (2917.9–5509.0)	3305.8 (2135.7–4629.7)	2889.2 (2118.5–3677.0)	347,354.1 (267,569.5–437,633.2)	276,598.4
Tunisia	2065.4 (1134.9–3084.8)	2248.3 (1594.2–2947.4)	2165.4 (1242.3–3352.4)	1889.1 (1269.8–2570.8)	217,846.2 (151,390.5–292,471.8)	272,878.3
United Arab Emirates	4028.4 (2596.4–5648.6)	4675.9 (3270.4–6389.7)	3425.1 (2209.4–4864.9)	3491.9 (2522.1–4661.1)	242,427.0 (171,328.5–322,519.2)	87,802.7
Yemen	2247.0 (881.5–4390.2)	3017.4 (1304.3–5529.8)	2373.0 (1016.2–4579.6)	3639.9 (1893.9–6356.4)	393,538.0 (216,892.2–664,897.8)	207,048.3

## Discussion

This is the first study to provide estimates of trends in obesity prevalence, deaths, and DALYs for the EMR from 1980 to 2015. Our study showed that the observed burden for high BMI was higher than expected for most countries in the region based on their SDI levels. Our study calls for renewed efforts to reduce the burden of obesity in the region. Indeed, with further progression of the epidemiologic transition and the growth and aging of the EMR population, high BMI will increase the burden of chronic conditions and disability and put financial and resource strains on the health systems.

High BMI is observed in some poor and rich countries of the EMR. In developed countries such as the United States and France, obesity is higher among low socioeconomic strata of the population (Drewnowski et al. 2014). These patterns in the West are attributable to poor diet and lower physical activity levels. Previous studies have reported similar findings for both socioeconomic status and sex in the EMR (Musaiger 2011a). In the EMR, except for Iran and Lebanon, all high-middle- and high-SDI countries had an obesity prevalence equaling or exceeding 25% for both males and females 20 years or older. Obesity prevalence exceeded 10% among children 2–19 years only in these countries as well, except for Bahrain, Jordan, Lebanon, and Iran. Djibouti and Egypt were the only low- and middle-SDI countries where childhood obesity exceeded 10% for females. These country-level estimates might be masking variations of the epidemic within each of the countries. Indeed, few studies have been done at the country level in the EMR, and these showed a variation in BMI levels between levels of education and income. High BMI was more likely to impact those with low educational levels (Sibai et al. 2003; Memish 2014).

Unfortunately, our estimations for observed obesity burden were higher than expected for the region, and based on SDI, which only deals with socioeconomic inequalities between countries. However, the biggest gaps between the included countries are cultural factors and obesogenic cultural traditions, as well as political instability (wars, civil unrest,) all of which are linked to obesity but not measured by SDI (Ulijaszek 2007). Cultural factors and obesogenic cultural traditions disproportionally affect women, highlighting cross-cutting gender issues in women’s health (Shapira 2013). This might explain the observed gender differences in overweight and obesity. Similarly, social determinants of health in countries in conflict tend to differ as morbidity and mortality are associated with conflict (World Health Organization 2008). More importantly, wars and civil unrest are closely associated with child malnutrition, which in turn is associated with increased risk of obesity, hypertension, cardiovascular disease, and type 2 diabetes (Devakumar et al. 2014; Charchuk et al. 2015). This leads to the intergenerational effects of war on obesity and the doubled burden of undernutrition in countries affected by wars or experiencing chronic civil unrest (Devakumar et al. 2014).

Despite this increase in obesity, EMR countries can hope to control their epidemic as declines in obesity prevalence have been reported in other countries due to health interventions and environmental and policy changes (Schmidt Morgen et al. 2013; Keane et al. 2014). EMR countries can benefit from implementing an array of proven interventions to control their obesity epidemics. Over the last decade, very few EMR ministries of health have focused on health promotion strategies to reduce obesity, such as awareness and behavioral changes. Of the 22 countries in the EMR, only Bahrain, Qatar, and Saudi Arabia had substantial information on combating obesity on their ministry of health websites (Gharib et al. 2012; Saudi Ministry of Health 2012). Qatar and Saudi Arabia launched national campaigns against obesity in 2011 and 2012, respectively. The Saudi campaign focused primarily on dietary awareness, promoting healthy eating choices and emphasizing variety and balance through a food pyramid (Hamad Al-Dkheel 2012). The Qatar campaign was more comprehensive, setting up programs to increase physical activity, replace school snacks with healthier options, and pilot a nutrition surveillance system in addition to a dietary awareness campaign (Mohammed Al-Thani 2011). Indeed, and despite the campaign in Qatar, obesity prevalence is one of the highest in the region, suggesting the need for a more aggressive approach.

Promoting healthier lifestyles around nutrition and physical activity is greatly needed in this region. As pointed out previously, possible factors determining obesity in the EMR include nutrition transition, inactivity, urbanization, marital status, a shorter duration of breastfeeding, frequent snacking, skipping breakfast, a high intake of sugary beverages, an increase in the incidence of eating outside the home, long periods of time spent viewing television, massive marketing promotion of high-fat foods, stunting, perceived body image, cultural elements, and food subsidy policies (Musaiger 2011b).

Indeed, estimates from the GBD study show an increase in dietary risk factors and low levels of physical activity (Forouzanfar et al. 2016). What the EMR populations consume can be directly affected by national policies, specifically those around food economics. For instance, in many EMR countries such as Egypt, Morocco, and Saudi Arabia, governments subsidize grains, bread, wheat flour, sugar, and cooking oil, and impose taxes on imported foods (Asfaw 2007; Musaiger 2011b). However, fruits and vegetables are neither subsidized nor exempt from import taxes, making healthier food choices harder for the population. As for physical activity, the lack of exercise facilities has been reported, at least in Egypt and Saudi Arabia, as the main reason for low physical activity (Al-Rafaee and Al-Hazzaa 2001; El-Gilany et al. 2011).

This study has a few limitations. These include possible underestimation of the prevalence of obesity or disease burden from high BMI as self-reported height and weight data were used. However, we corrected these based on measured data at each age, sex, and country unit (GBD 2015 Obesity Collaborators 2017). Briefly, no significant difference between measured and self-reported data for children was found. For adults, self-reported data were adjusted for overweight prevalence, obesity prevalence, and mean BMI using nested hierarchical mixed-effects regression models, fit using restricted maximum likelihood separately by sex. Second, estimates for some countries with sparse data were driven by covariates in the statistical modeling. This, in fact, highlights the need for more timely surveillance data on BMI and risk factors in the EMR. Better quality data in the EMR for quantification of morbidity and mortality burden and health policy planning is urgently needed. Third, the attributable effect of BMI on ischemic heart disease, stroke, and diabetes was derived from prospective observational studies and meta-analyses, and hence does not account for differences by ethnicity, or for underlying diseases (Global BMI Mortality Collaboration et al. 2016). While these observational studies are not specifically from the EMR, the attributable effect of BMI on the three diseases has been shown to remain the same across different regions from the world (Singh et al. 2013). Fourth, BMI presents some drawbacks as it is a measure of excess weight rather excess body fat (Daniels 2009). The relationship between BMI and body fat can be influenced by age, sex, ethnicity, and muscle mass. In addition, BMI does not indicate whether excess weight is due to fat, muscle, or bone (Daniels 2009). However, BMI is favored for its ease of use in surveys and is the most available indicator regarding excess weight. More details on this study’s limitations are available elsewhere (GBD 2015 Obesity Collaborators 2017).

This study showed that high BMI creates a major burden in the EMR. We call for countries in the region to invest more resources in prevention and health promotion efforts to reduce the prevalence of obesity. These programs should focus on stopping weight gain as a first step and more aggressive programs to reduce weight among those who need to do so. These programs should take into account the culture and local environment. Moreover, countries should join efforts in their programs and policies and share experiences and success stories.

## Electronic supplementary material

Below is the link to the electronic supplementary material.

Supplementary material 1 (XLSX 20 kb)

Supplementary material 2 (DOCX 23 kb)

## References

[CR1] Abou Abbas L, Salameh P, Nasser W (2015). Obesity and symptoms of depression among adults in selected countries of the Middle East: a systematic review and meta-analysis. Clin Obes.

[CR4] Al-Rafaee SA, Al-Hazzaa HM (2001). Physical activity profile of adult males in Riyadh city. Saudi Med J.

[CR5] Asfaw A (2007). Do government food price policies affect the prevalence of obesity? Empirical evidence from Egypt. World Dev.

[CR6] Broyles ST, Denstel KD, Church TS (2015). The epidemiological transition and the global childhood obesity epidemic. Int J Obes Suppl.

[CR7] Charchuk R, Houston S, Hawkes MT (2015). Elevated prevalence of malnutrition and malaria among school-aged children and adolescents in war-ravaged South Sudan. Pathog Glob Health.

[CR8] Cole TJ, Bellizzi MC, Flegal KM, Dietz WH (2000). Establishing a standard definition for child overweight and obesity worldwide: international survey. BMJ.

[CR9] Daniels SR (2009). The use of BMI in the clinical setting. Pediatrics.

[CR10] Devakumar D, Birch M, Osrin D (2014). The intergenerational effects of war on the health of children. BMC Med.

[CR11] Drewnowski A, Moudon AV, Jiao J (2014). Food environment and socioeconomic status influence obesity rates in Seattle and in Paris. Int J Obes.

[CR12] El-Gilany AH, Badawi K, El-Khawaga G, Awadalla N (2011). Physical activity profile of students in Mansoura University, Egypt. East Mediterr Health J Rev Sante Mediterr Orient Al-Majallah Al-Sihhiyah Li-Sharq Al-Mutawassit.

[CR13] Forouzanfar MH, Alexander L, Anderson HR (2015). Global, regional, and national comparative risk assessment of 79 behavioural, environmental and occupational, and metabolic risks or clusters of risks in 188 countries, 1990–2013: a systematic analysis for the Global Burden of Disease Study 2013. Lancet.

[CR14] Forouzanfar MH, Afshin A, Alexander LT (2016). Global, regional, and national comparative risk assessment of 79 behavioural, environmental and occupational, and metabolic risks or clusters of risks, 1990–2015: a systematic analysis for the Global Burden of Disease Study 2015. Lancet.

[CR2] GBD 2015 Obesity Collaborators (2017) Health effects of overweight and obesity in 195 Countries over 25 Years. N Engl J Med 377:13–27. doi:10.1056/NEJMoa1614362 10.1056/NEJMoa1614362 PMC5477817 28604169

[CR15] Gharib N, Al Amer M, Al-Salehi S (2012) Nutrition clinics: management and prevention of obesity. http://hajj.moh.gov.bh/pdf/publications/X_217201413620.pdf. Accessed 27 Apr 2017

[CR16] Di Angelantonio E, Bhupathiraju S, Global BMI Mortality Collaboration null (2016). Body-mass index and all-cause mortality: individual-participant-data meta-analysis of 239 prospective studies in four continents. Lancet Lond Engl.

[CR17] Hamad Al-Dkheel M (2012) Dietary Guidelines for Saudis: the healthy food palm. http://www.moh.gov.sa/en/Ministry/MediaCenter/Publications/Documents/final%20english%20%20%D8%A7%D9%84%D9%83%D8%AA%D8%A7%D8%A8%20%D8%A7%D9%84%D8%B9%D9%84%D9%85%D9%8A%20%D8%A5%D9%86%D8%AC%D9%84%D9%8A%D8%B2%D9%8A.pdf. Accessed 27 Apr 2017

[CR18] Hill AB (1965). The environment and disease: association or causation?. Proc R Soc Med.

[CR19] Institute for Health Metrics and Evaluation (2016) Global Burden of Disease Study 2015 (GBD 2015) Data Resources | GHDx. http://ghdx.healthdata.org/gbd-2015. Accessed 30 May 2017

[CR20] Keane E, Kearney PM, Perry IJ (2014). Trends and prevalence of overweight and obesity in primary school aged children in the Republic of Ireland from 2002–2012: a systematic review. BMC Public Health.

[CR21] Kelly SP, Graubard BI, Andreotti G (2017). Prediagnostic body mass index trajectories in relation to prostate cancer incidence and mortality in the PLCO cancer screening trial. J Natl Cancer Inst.

[CR22] Kim SH, Després J-P, Koh KK (2016). Obesity and cardiovascular disease: friend or foe?. Eur Heart J.

[CR23] Maciosek MV, LaFrance AB, Dehmer SP (2017). Updated priorities among effective clinical preventive services. Ann Fam Med.

[CR24] Mehta NK, Patel SA, Ali MK, Venkat Narayan KM (2017). Preventing disability: the influence of modifiable risk factors on state and national disability prevalence. Health Aff Proj Hope.

[CR25] Memish ZA (2014). Obesity and associated factors—Kingdom of Saudi Arabia, 2013. Prev Chronic Dis.

[CR26] Mohammed Al-Thani AA (2011) Qatar national nutrition and physical activity action plan 2011–2016. https://extranet.who.int/nutrition/gina/sites/default/files/QAT%202011%20National%20Nutrition%20and%20Physical%20Activity%20Action%20Plan.pdf. Accessed 27 Apr 2017

[CR27] Mokdad AH, Forouzanfar MH, Daoud F (2016). Health in times of uncertainty in the eastern Mediterranean region, 1990–2013: a systematic analysis for the Global Burden of Disease Study 2013. Lancet Glob Health.

[CR28] Musaiger AO (2011a) Food consumption patterns in the Eastern Mediterranean Region. https://www.researchgate.net/publication/236668592_Food_Consumption_Patterns_in_the_Eastern_Mediterranean_Region. Accessed 27 Apr 2017

[CR29] Musaiger AO (2011). Overweight and obesity in eastern mediterranean region: prevalence and possible causes. J Obes.

[CR30] Musaiger AO, Al-Hazzaa HM, Takruri HR, Mokhatar N (2012). Change in nutrition and lifestyle in the Eastern Mediterranean Region: health Impact. J Nutr Metab.

[CR31] Ng M, Fleming T, Robinson M (2014). Global, regional, and national prevalence of overweight and obesity in children and adults during 1980–2013: a systematic analysis for the Global Burden of Disease Study 2013. Lancet.

[CR32] Ng M, Liu P, Thomson B, Murray CJL (2016). A novel method for estimating distributions of body mass index. Popul Health Metr.

[CR33] Pratt LA, Brody DJ (2014) Depression and obesity in the U.S. adult household population, 2005–2010. NCHS Data Brief 167:1–8 25321386

[CR34] Saudi Ministry of Health (2012) National campaign against overweight and obesity. http://www.moh.gov.sa/en/. Accessed 7 Apr 2017

[CR35] Schmidt Morgen C, Rokholm B, Sjöberg Brixval C (2013). Trends in prevalence of overweight and obesity in danish infants, children and adolescents—are we still on a plateau?. PLoS One.

[CR36] Shapira N (2013). Women’s higher health risks in the obesogenic environment: a gender nutrition approach to metabolic dimorphism with predictive, preventive, and personalised medicine. EPMA J.

[CR37] Sibai AM, Hwalla N, Adra N, Rahal B (2003). Prevalence and covariates of obesity in lebanon: findings from the first epidemiological study. Obes Res.

[CR38] Sidney S, Quesenberry CP, Jaffe MG (2016). Recent trends in cardiovascular mortality in the United States and public health goals. JAMA Cardiol.

[CR39] Singh GM, Danaei G, Farzadfar F (2013). The age-specific quantitative effects of metabolic risk factors on cardiovascular diseases and diabetes: a pooled analysis. PLoS One.

[CR40] Stevens GA, Alkema L, Black RE (2016). Guidelines for accurate and transparent health estimates reporting: the GATHER statement. Lancet.

[CR41] Terkawi AS, Rafiq M, Algadaan R (2014). General anesthesia for the heaviest man in the world. Saudi J Anaesth.

[CR42] Ulijaszek SJ (2007). Frameworks of population obesity and the use of cultural consensus modeling in the study of environments contributing to obesity. Econ Hum Biol.

[CR43] Wang H, Naghavi M, Allen C (2016). Global, regional, and national life expectancy, all-cause mortality, and cause-specific mortality for 249 causes of death, 1980–2015: a systematic analysis for the Global Burden of Disease Study 2015. Lancet.

[CR44] World Cancer Research Fund, American Institute for Cancer Research (2007). Food, nutrition, physical activity, and the prevention of cancer: a global perspective.

[CR45] World Health Organization (2008) Integrated health services—what and why? http://www.who.int/healthsystems/technical_brief_final.pdf. Accessed 27 Apr 2017

[CR46] World Health Organization (2016) Eastern Mediterranean Region office. About us. http://www.emro.who.int/entity/about-us/index.html. Accessed 27 Apr 2017

[CR47] Yao T-C, Tsai H-J, Chang S-W (2017). Obesity disproportionately impacts lung volumes, airflow and exhaled nitric oxide in children. PLoS One.

