# Supplementary material for: Burden of obesity in the Eastern Mediterranean Region: findings from the Global Burden of Disease 2015 study
Source: Int J Public Health. 2017 Aug 3;63(Suppl 1):165–76. doi: 10.1007/s00038-017-1002-5 (PMC5973977; doi:10.1007/s00038-017-1002-5)
Supplement: Supplementary file 2 — Supplementary material 2 (DOCX 23 kb) [file 38_2017_1002_MOESM2_ESM.docx]

Electronic Supplementary Material

**Article title:**

Burden of obesity in the Eastern Mediterranean Region: findings from the Global Burden of Disease 2015 study

**Journal:**

International Journal of Public Health

**Authors:**

GBD 2015 Eastern Mediterranean Region Obesity Collaborators

**Corresponding author:**

Ali H. Mokdad

Institute for Health Metrics and Evaluation, University of Washington, Seattle, WA, United States

Email: [mokdaa@uw.edu](mailto:mokdaa@uw.edu)

E-Table 1: Prevalence of overweight and obesity, with 95% uncertainty intervals (UI) among adults aged 20 years or older (Global Burden of Disease 2015 study, Eastern Mediterranean countries, 1980 and 2015)

|  |  | 1980 | | | | 2015 | | | |
| --- | --- | --- | --- | --- | --- | --- | --- | --- | --- |
|  |  | Overweight | | Obesity | | Overweight | | Obesity | |
| Country | Socio-demographic Index (SDI) | Males | Females | Males | Females | Males | Females | Males | Females |
| Afghanistan | Low SDI | 29.9  (27.4 – 32.6) | 27.3  (24.9 – 29.3) | 12.7  (11.0 – 14.6) | 17.7  (15.6 – 20.1) | 23.1  (20.9 – 25.4) | 22.0  (21.1 – 23.0) | 7.9  (6.8 – 9.0) | 12.1  (11.1 – 13.1) |
| Bahrain | High-middle SDI | 35.8  (33.4 – 37.9) | 31.3  (29.7 – 32.6) | 18.6  (16.5 – 20.7) | 31.0  (28.0 – 34.0) | 38.1  (36.7 – 38.9) | 32.0  (31.3 – 32.6) | 28.1  (25.8 – 30.7) | 38.2  (35.3 – 41.2) |
| Djibouti | Low-middle SDI | 9.7  (7.3 – 12.2) | 13.2  (11.2 – 15.2) | 2.3  (1.3 – 3.8) | 3.3  (2.4 – 4.5) | 25.3  (22.0 – 28.3) | 27.1  (25.5 – 28.5) | 9.8  (6.4 – 13.8) | 19.4  (15.8 – 23.3) |
| Egypt | Middle SDI | 34.2  (31.3 – 36.9) | 33.1  (31.5 – 34.8) | 11.2  (9.8 – 12.8) | 23.8  (21.3 – 26.4) | 41.8  (40.6 – 43.1) | 32.4  (32.1 – 32.6) | 27.0  (24.7 – 29.3) | 43.0  (40.3 – 45.6) |
| Iran | High-middle SDI | 23.9  (21.0 – 26.7) | 31.7  (29.4 – 33.8) | 5.8  (4.9 – 6.8) | 16.6  (14.6 – 18.8) | 35.3  (33.0 – 37.5) | 32.3  (30.5 – 34.0) | 13.8  (12.5 – 15.1) | 24.1  (21.9 – 26.2) |
| Iraq | Middle SDI | 36.7  (34.6 – 38.8) | 30.7  (29.6 – 32.0) | 23.2  (20.9 – 25.5) | 33.9  (30.8 – 36.8) | 37.2  (36.0 – 38.8) | 30.6  (29.7 – 31.2) | 25.8  (23.5 – 28.0) | 37.0  (34.0 – 39.9) |
| Jordan | High-middle SDI | 36.4  (33.8 – 38.7) | 31.4  (29.5 – 32.7) | 14.8  (13.0 – 16.6) | 27.2  (24.6 – 29.9) | 41.6  (40.6 – 42.6) | 30.1  (30.0 – 30.3) | 31.8  (29.3 – 34.1) | 43.4  (40.9 – 45.8) |
| Kuwait | High SDI | 29.6 (27.7 – 31.1) | 33.8 (33.0 – 34.7) | 23.0 (20.5 – 25.5) | 38.9 (36.1 – 41.6) | 37.0 (37.1 – 37.0) | 32.4 (32.5 – 32.1) | 41.4 (39.1 – 43.5) | 49.5 (47.8 – 51.4) |
| Lebanon | High-middle SDI | 33.7 (30.7 – 36.8) | 30.5 (27.8 – 33.1) | 9.2 (8.0 – 10.5) | 12.8 (11.1 – 14.6) | 42.6 (40.8 – 43.9) | 33.1 (31.7 – 34.4) | 22.2 (20.2 – 24.1) | 24.6 (22.4 – 27.0) |
| Libya | Middle SDI | 38.1 (35.8 – 40.8) | 32.2 (30.8 – 33.5) | 16.9 (14.8 – 18.9) | 27.7 (25.0 – 30.7) | 39.6 (37.6 – 41.3) | 32.3 (31.2 – 33.1) | 21.8 (19.6 – 24.2) | 34.9 (31.9 – 38.2) |
| Morocco | Low-middle SDI | 24.74 (22.0 – 27.7) | 24.8 (22.7 – 27.2) | 6.0 (5.1 – 7.0) | 11.4 (9.7 – 13.1) | 37.2 (34.3 – 40.0) | 32.4 (30.5 – 33.9) | 11.8 (10.4 – 13.3) | 23.1 (20.6 – 25.7) |
| Oman | High-middle SDI | 27.0 (24.3 – 29.9) | 29.0 (26.8 – 30.8) | 9.2 (7.9 – 10.5) | 20.2 (18.2 – 22.6) | 39.6 (38.1 – 40.9) | 31.8 (31.0 – 32.4) | 24.8 (22.6 – 27.1) | 37.9 (35.2 – 40.8) |
| Pakistan | Low-middle SDI | 16.8 (14.1 – 20.0) | 21.3 (17.6 – 24.7) | 3.6 (2.7 – 4.7) | 7.0 (5.5 – 8.8) | 24.3 (21.2 – 27.3) | 24.4 (22.6 – 26.4) | 7.3 (5.9 – 9.1) | 14.5 (12.3 – 16.9) |
| Palestine | Middle SDI | 27.0 (24.3 – 29.8) | 32.4 (30.5 – 34.6) | 10.1 (8.8 – 11.6) | 21.0 (18.6 – 23.2) | 38.6 (37.5 – 39.6) | 37.2 (36.6 – 37.4) | 24.5 (22.4 – 26.6) | 38.1 (35.6 – 40.5) |
| Qatar | High-middle SDI | 39.1 (39.2 – 39.2) | 18.6 (18.1 – 19.4) | 43.5 (40.6 – 46.2) | 53.0 (49.4 – 56.2) | 36.8 (36.7 – 36.8) | 23.6 (23.7 – 23.6) | 42.5 (40.1 – 44.8) | 52.4 (50.3 – 54.6) |
| Saudi Arabia | High-middle SDI | 32.4 (31.3 – 33.2) | 32.0 (31.1 – 32.5) | 11.4 (11.0 – 12.0) | 26.5 (25.7 – 27.5) | 38.8 (38.3 – 39.2) | 30.3 (30.2 – 30.5) | 29.8 (28.9 – 30.7) | 43.1 (42.1 – 44.1) |
| Somalia | Low SDI | 9.4 (7.2 – 11.7) | 18.0 (15.7 – 20.2) | 2.5 (1.4 – 4.2) | 8.4 (6.5 – 10.7) | 10.7 (8.3 – 13.2) | 19.4 (17.6 – 21.1) | 2.5 (1.5 – 4.0) | 10.5 (8.3 – 12.8) |
| Sudan | Low-middle SDI | 19.7 (17.4 – 22.2) | 28.0 (26.0 – 29.9) | 5.5 (4.6 – 6.5) | 18.2 (16.0 – 20.5) | 28.6 (26.1 – 31.1) | 30.3 (28.7 – 31.6) | 11.5 (10.0 – 13.1) | 28.3 (25.6 – 31.2) |
| Syria | Middle SDI | 38.5 (36.1 – 41.1) | 34.3 (32.4 – 36.0) | 14.2 (12.6 – 15.9) | 23.6 (21.3 – 26.0) | 43.1 (40.9 – 45.0) | 35.1 (33.7 – 36.2) | 19.9 (17.9 – 22.0) | 30.2 (27.6 – 33.0) |
| Tunisia | Middle SDI | 27.2 (24.5 – 30.5) | 29.0 (26.6 – 31.6) | 5.4 (4.5 – 6.3) | 13.6 (11.8 – 15.5) | 40.1 (37.2 – 42.4) | 33.4 (31.6 – 35.1) | 12.1 (10.7 – 13.8) | 23.3 (20.8 – 25.8) |
| United Arab Emirates | High SDI | 38.5 (37.2 – 39.9) | 28.9 (27.6 – 30.1) | 25.2 (22.6 – 27.5) | 31.2 (28.1 – 34.1) | 38.1 (36.4 – 39.8) | 31.4 (30.3 – 32.5) | 25.7 (23.2 – 28.2) | 31.6 (28.9 – 34.6) |
| Yemen | Low-middle SDI | 13.5 (11.6 – 15.6) | 19.91 (17.3 – 22.7) | 1.9 (1.6 – 2.3) | 4.8 (3.9 – 5.8) | 24.5 (21.9 – 27.3) | 25.4 (23.9 – 26.8) | 5.3 (4.5 – 6.3) | 12.8 (11.5 – 14.2) |

E-Table 2: Prevalence of overweight and obesity, with 95% uncertainty intervals (UI), among children aged 2–19 years (Global Burden of Disease 2015 study, Eastern Mediterranean countries, 1980 and 2015)

|  |  | 1980 | | | | 2015 | | | |
| --- | --- | --- | --- | --- | --- | --- | --- | --- | --- |
|  |  | Overweight | | Obesity | | Overweight | | Obesity | |
| Country | Socio-demographic Index (SDI) | Males | Females | Males | Females | Males | Females | Males | Females |
| Afghanistan | Low SDI | 7.9  (5.8 – 10.2) | 14.1  (11.2 – 17.3) | 4.0  (2.8 – 5.6) | 6.2  (4.7 – 8.2) | 4.9  (3.7 – 6.3) | 9.3  (7.2 – 11.6) | 2.1  (1.5 – 3.0) | 4.1  (2.9 – 5.5) |
| Bahrain | High-middle SDI | 11.2  (8.7 – 14.1) | 15.9  (12.9 – 19.1) | 7.8  (5.6 – 10.2) | 9.3  (7.0 – 12.2) | 12.0  (9.4 – 14.9) | 12.6  (9.9 – 15.7) | 7.7  (5.6 – 10.4) | 7.8  (5.9 – 10.4) |
| Djibouti | Low-middle SDI | 4.8  (3.1 – 7.3) | 8.8  (6.1 – 11.4) | 2.1  (1.0 – 3.7) | 3.6  (2.1 – 5.7) | 11.6  (8.2 – 15.6) | 15.5  (12.5 – 19.4) | 7.4  (3.9 – 11.6) | 10.5  (6.9 – 15.1) |
| Egypt | Middle SDI | 10.0  (7.5 – 12.9) | 15.1  (11.8 – 18.8) | 2.8  (1.9 – 3.8) | 4.3  (3.1 – 5.9) | 17.1  (13.6 – 20.6) | 18.3  (15.3 – 22.0) | 9.4  (6.9 – 12.2) | 11.0  (8.4 – 13.9) |
| Iran | High-middle SDI | 4.1  (3.1 – 5.4) | 6.4  (4.7 – 8.4) | 1.2  (0.8 – 1.7) | 1.7  (1.2 – 2.3) | 8.9  (6.8 – 11.1) | 10.3  (7.7 – 12.7) | 4.3  (3.0 – 5.7) | 4.5  (3.2 – 6.1) |
| Iraq | Middle SDI | 8.6  (6.6 – 11.2) | 11.4  (8.9 – 14.4) | 3.7  (2.6 – 5.0) | 4.2  (3.0 – 5.7) | 9.9  (7.8 – 12.4) | 12.7  (10.0 – 15.3) | 5.6  (4.1 – 7.5) | 5.8  (4.3 – 7.7) |
| Jordan | High-middle SDI | 6.3  (4.6 – 8.3) | 9.6  (7.3 – 12.3) | 1.6  (1.1 – 2.2) | 2.3  (1.6 – 3.2) | 13.0  (10.2 – 16.3) | 15.4  (12.7 – 18.5) | 6.3  (4.6 – 8.2) | 6.0  (4.6 – 7.8) |
| Kuwait | High SDI | 12.2 (9.6 – 15.3) | 15.6 (13.0 – 18.6) | 11.0 (8.1 – 14.0) | 11.0 (8.4 – 13.9) | 19.1 (16.2 – 21.8) | 20.6 (17.5 – 23.6) | 22.1 (17.8 – 27.0) | 19.2 (15.2 – 23.4) |
| Lebanon | High-middle SDI | 8.1 (6.2 – 10.4) | 10.2 (8.0 – 12.4) | 4.1 (2.9 – 5.6) | 3.7 (2.6 – 5.0) | 13.1 (10.1 – 15.9) | 14.1 (11.4 – 17.4) | 7.5 (5.5 – 9.7) | 6.4 (4.8 – 8.5) |
| Libya | Middle SDI | 12.8 (9.8 – 15.6) | 17.3 (14.2 – 20.6) | 7.5 (5.5 – 10.0) | 8.7 (6.4 – 11.2) | 13.7 (10.9 – 16.7) | 17.8 (14.4 – 21.3) | 8.8 (6.6 – 11.4) | 9.8 (7.4 – 12.5) |
| Morocco | Low-middle SDI | 6.4 (4.8 – 8.5) | 10.4 (8.0 – 13.4) | 2.1 (1.5 – 2.9) | 2.6 (1.9 – 3.6) | 10.8 (8.3 – 13.9) | 13.3 (10.4 – 16.3) | 4.7 (3.3 – 6.5) | 4.9 (3.5 – 6.5) |
| Oman | High-middle SDI | 5.9 (4.3 – 7.9) | 10.7 (8.5 – 13.2) | 2.6 (1.8 – 3.6) | 5.5 (4.0 – 7.4) | 16.2 (12.7 – 19.5) | 16.4 (13.4 – 19.4) | 11.6 (8.6 – 14.9) | 12.0 (9.1 – 15.3) |
| Pakistan | Low-middle SDI | 5.5 (3.9 – 7.8) | 5.8 (4.1 – 7.6) | 3.0 (2.0 – 4.5) | 3.0 (2.0 – 4.5) | 3.4 (2.3 – 4.7) | 5.3 (3.8 – 7.7) | 2.0 (1.2 – 2.9) | 2.2 (1.4 – 3.3) |
| Palestine | Middle SDI | 7.0 (5.3 – 9.3) | 9.3 (7.1 – 11.9) | 2.0 (1.4 – 2.7) | 2.3 (1.6 – 3.2) | 11 (8.5 – 14.1) | 12.3 (9.4 – 15.3) | 4.6 (3.3 – 6.1) | 4.9 (3.5 – 6.5) |
| Qatar | High-middle SDI | 14.7 (12.2 – 17.1) | 9.0 (7.2 – 10.8) | 20.5 (16.1 – 25.3) | 10.6 (7.9 – 13.8) | 16.1 (13.3 – 18.7) | 13.4 (11 – 16.0) | 20.8 (16.6 – 25.2) | 13.5 (10.3 – 17.2) |
| Saudi Arabia | High-middle SDI | 6.1 (5.5 – 6.8) | 11.1 (10.2 – 12.0) | 2.6 (2.2 – 3.0) | 4.5 (4.0 – 5.1) | 15.3 (14.2 – 16.5) | 19.3 (18.0 – 20.5) | 11.1 (10.1 – 12.2) | 13.8 (12.5 – 15.2) |
| Somalia | Low SDI | 4.5 (2.8 – 6.4) | 6.3 (4.5 – 8.1) | 2.6 (1.3 – 4.8) | 2.8 (1.7 – 4.4) | 4.3 (2.7 – 6.2) | 7.0 (5.0 – 9.3) | 2.5 (1.2 – 4.3) | 3.2 (2.0 – 4.8) |
| Sudan | Low-middle SDI | 2.9 (2.1 – 3.7) | 5.4 (4.1 – 6.7) | 1.5 (1.0 – 2.2) | 2.2 (1.5 – 3.2) | 3.3 (2.4 – 4.4) | 5.3 (4.0 – 6.9) | 2.1 (1.5 – 3.0) | 2.7 (2.0– 3.6) |
| Syria | Middle SDI | 10.0 (7.6 – 12.5) | 12.3 (9.6 – 15.2) | 3.6 (2.4 – 4.9) | 5.3 (3.8 – 7.0) | 13.3 (10.7 – 16.4) | 13.0 (10.5 – 16.0) | 7.4 (5.4 – 9.8) | 6.3 (4.6 – 8.3) |
| Tunisia | Middle SDI | 6.0 (4.4 – 8.0) | 7.4 (5.7 – 9.3) | 1.4 (1.0 – 2.0) | 2.1 (1.4 – 2.8) | 13.0 (9.9 – 16.5) | 13.5 (10.7 – 16.6) | 4.1 (2.9 – 5.5) | 4.2 (3.1 – 5.8) |
| United Arab Emirates | High SDI | 17.2 (13.9 – 20.7) | 18.1 (14.2 – 21.7) | 10.5 (8.0 – 13.4) | 9.7 (7.3 – 12.7) | 16.3 (12.9 – 19.5) | 17.6 (14.0 – 21.6) | 11.3 (8.5 – 14.2) | 10.3 (7.9 – 13.3) |
| Yemen | Low-middle SDI | 2.7 (2.0 – 3.6) | 9.3 (7.1 – 11.6) | 0.5 (0.3 – 0.6) | 2.5 (1.7 – 3.4) | 5.1 (3.8 – 6.7) | 8.6 (6.6 – 11.0) | 1.3 (0.9 – 1.8) | 3.7 (2.6 – 5.2) |
